# Analysis of the twenty-six largest outbreaks of tuberculosis in Aragon using whole-genome sequencing for surveillance purposes

**DOI:** 10.1038/s41598-022-23343-1

**Published:** 2022-11-05

**Authors:** Jessica Comín, Alberto Cebollada, Daniel Ibarz, Jesús Viñuelas, Juan Sahagún, Luis Torres, María-José Iglesias, Sofía Samper

**Affiliations:** 1 Instituto Aragonés de Ciencias de la Salud, C/de San Juan Bosco, 13, 50009 Zaragoza, Spain; 2 Universidad de Zaragoza, C/Domingo Miral S/N, 50009 Zaragoza, Spain; 3 Hospital Universitario Miguel Servet, Paseo Isabel la Católica, 1-3, 50009 Zaragoza, Spain; 4 Grupo de Estudio de Infecciones por Micobacterias (GEIM), Sociedad Española de Enfermedades Infecciosas y Microbiología Clínica, C/Agustín de Bentacourt, No 13, 28003 Madrid, Spain; 5 Hospital Clínico Universitario Lozano Blesa, C/ de San Juan Bosco, 15, 50009 Zaragoza, Spain; 6 Hospital San Jorge, Av. Martínez de Velasco, 36, 22004 Huesca, Spain; 7 Fundación IIS Aragón, C/de San Juan Bosco, 13, 50009 Zaragoza, Spain; 8 CIBER de Enfermedades Respiratorias, Av. Monforte de Lemos, 3-5. Pabellón 11, Planta 0, 28029 Madrid, Spain

**Keywords:** Infectious-disease epidemiology, Bacterial genomics, Pathogens

## Abstract

The incidence of tuberculosis in Aragon, Spain, is around ten cases per 100,000 inhabitants. Since 2004, a molecular surveillance protocol has been carried out; therefore, all *M. tuberculosis* strains are genotyped. Recently, whole-genome sequencing has been implemented for relevant isolates. The aim of this work is to characterise at the molecular level the causative strains of the 26 largest outbreaks of the community (including ten or more cases), genotyped by IS*6110*-RFLP and causing 26% of tuberculosis cases. To achieve this objective, two or three isolates of each IS*6110*-cluster belonging to different years were selected for sequencing. We found that strains of lineages L4.8, L4.3 and L4.1.2 were the most frequent. The threshold of 12 SNPs as the maximum distance for confirming the belonging to an outbreak was met for 18 of the 26 IS*6110*-clusters. Four pairs of isolates with more than 90 SNPs were identified as not belonging to the same strain, and four other pairs were kept in doubt as the number of SNPs was close to 12, between 14 and 35. The study of Regions of Difference revealed that they are lineage conserved. Moreover, we could analyse the IS*6110* locations for all genome-sequenced isolates, finding some frequent locations in isolates belonging to the same lineage and certain IS*6110* movements between the paired isolates. In the vast majority, these movements were not captured by the IS*6110*-RFLP pattern. After classifying the genes containing SNP by their functional category, we could confirm that the number of SNPs detected in genes considered as virulence factors and the number of cases the strain produced were not related, suggesting that a particular SNP is more relevant than the number. The characteristics found in the most successful strains in our community could be useful for other researchers in epidemiology, virulence and pathogenesis.

## Introduction

Tuberculosis (TB) is the world’s leading infectious disease killer, just surpassed by COVID-19 in 2020. In 2019, 10 million people fell ill with TB and 1.2 million died because of it^1^. The causative agent is *Mycobacterium tuberculosis*, with pulmonary TB being the most frequent presentation of the disease, although extrapulmonary forms can also occur^2^.

*M. tuberculosis* belongs to the *M. tuberculosis* complex (MTBC), which includes eight phylogenetic lineages. L1, L5, L6 and L7 are considered ancient lineages, along with the animal branch, while L2, L3 and L4 are considered modern lineages^3^. Members of L2 and L4 are responsible for the majority of TB cases in the world, and particularly, L4 and its corresponding sub-lineages are the most widespread among our population^4^.

Since 2004, a TB surveillance protocol has been carried out in Aragon, Spain, a low-incidence country with around ten cases per 100,000 inhabitants. All *M. tuberculosis* isolates are genotyped by IS*6110*-RFLP and Spoligotyping. As a result, we have a register of all *M. tuberculosis* genotypes and their relatedness (i.e. their belonging to some outbreak).

With the development of whole-genome sequencing (WGS), this technique is proposed to replace the standard molecular typing techniques as WGS has the highest resolution power^5^ and is now becoming affordable for investigation laboratories^6^.

To implement WGS in our routine laboratory, we planned to sequence two or three representative isolates of the 26 largest outbreaks in our community, which contained at least ten cases. The aim of this work is to characterise these strains at the molecular level and get a general view of the properties of these successful strains to be considered in future surveillance protocols.

## Results

**Figure 1:**
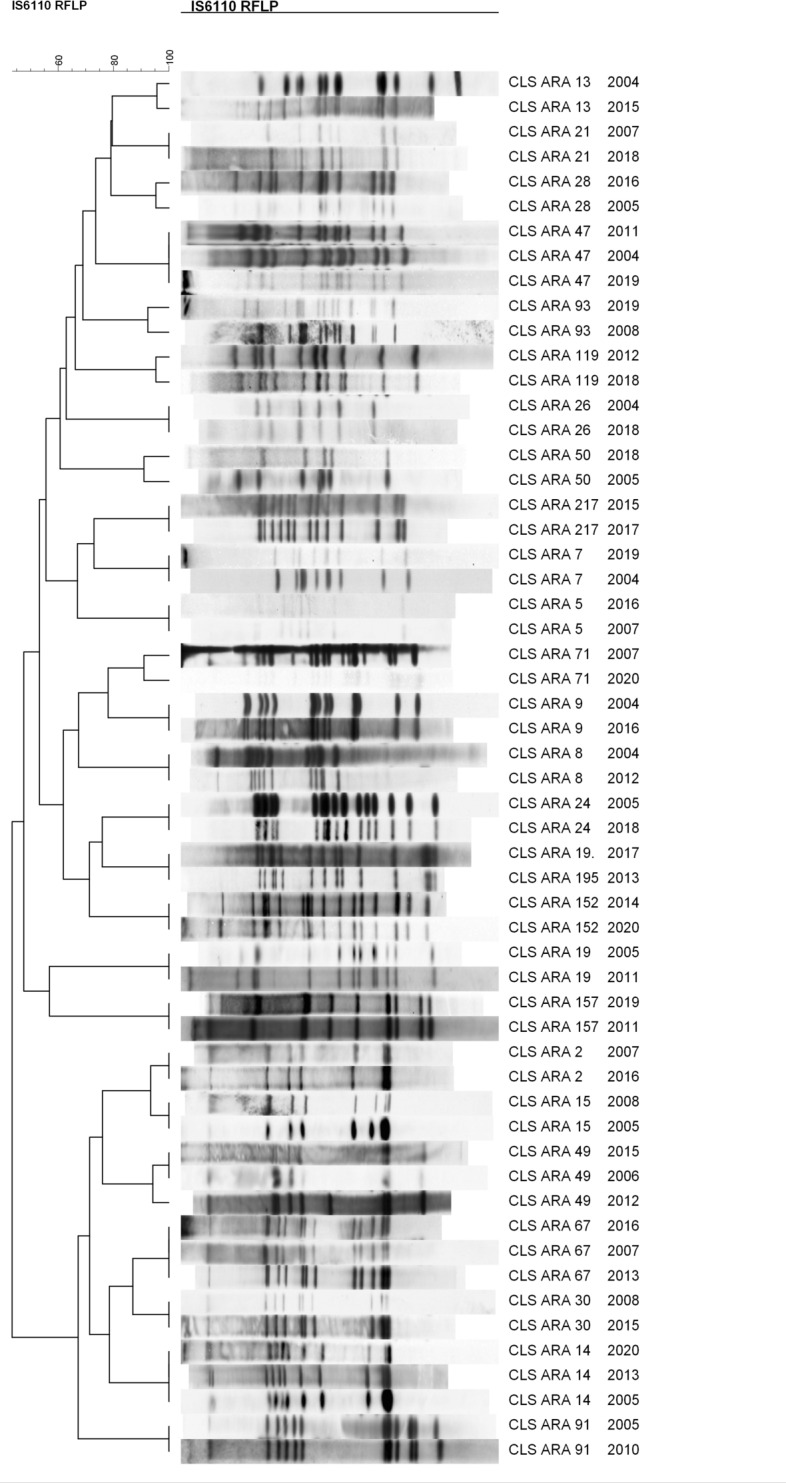
Dendrogram showing the IS*6110*-RFLP patterns of the selected isolates of the different outbreaks.

With the aim of characterising at the molecular level the responsible strains of the largest outbreaks in our community, comprising 665 out of the 2553 cases registered, we sequenced the genomes of the representative IS*6110*-clustered strains. The dendrogram based on their IS*6110*-RFLP patterns is shown in Fig. 1, and the MIRU-VNTR patterns and spoligotypes are detailed in Table S1.

### General views

The 26 IS*6110*-outbreaks studied ranged from 10 to 178 cases since 2004. Seven outbreaks were caused by L4.8 strains producing 291 cases. These include CLS_7^7^, the largest outbreak we ever had, with 242 cases from 1993 to 2020 (no data for 1996–2000). Eight outbreaks were caused by L4.1.2/Haarlem strains producing 170 cases. Six outbreaks were produced by L4.3/LAM strains (two by a L4.3.2 strain, three by a L4.3.3 strain and one by a L4.3.4 strain), producing 121 cases. These included CLS_217, which was independently studied^8^. The rest of the outbreaks were caused by strains from different lineages: one by a L4.1.1.3/X strain, with 21 cases^9^; one by a L4.7 strain, with 19 cases; one by a L4.4.1.1 strain, with 16 cases; one by a L4.9 strain, producing 13 cases; and one by a L4.6.1.1 strain, with 14 cases.

### SNP distances

It has been proposed that a distance of ≤ 5 SNPs between two isolates is considered recent contact and that 12 SNPs should be the maximum distance to consider both isolates to be the same strain and therefore the same outbreak^10^. Eighteen out of the 26 IS*6110*-clusters studied fit this threshold, indicating that the sequenced isolates belong to the same WGS-outbreak. We found a distance of 1–10 SNPs among the two or three isolates sequenced for 18 of the IS*6110*-clusters studied (Table 1). In Table S2, a description of the SNPs found in these clusters (point, gene, type of mutation, effect of the mutation) can be found. For CLS_15, lineage identification revealed that one selected isolate belonged to L4.1.2.1 and the other to L4.8. A new revision of the genotype patterns confirmed that the correct one was L4.1.2.1, so the L4.8 isolate was a selection error. The SNP distance could not be studied for this IS*6110*-cluster. For three clusters, the SNP distance was large enough to guarantee that the selected isolates were not the same strain. CLS_2 had an SNP distance of 145, CLS_49 of 143 among the three sequenced isolates, and CLS_26 of 93. On the other hand, four clusters had a distance higher than 12 SNPs but close. CLS 157 had a distance of 34 SNPs between the two sequenced isolates. CLS_47 had an SNP distance of 35 among the three sequenced isolates; however, the majority of SNPs were due to one of the isolates, with the other two being more related (less than 12 SNPs). Finally, CLS_119 had an SNP distance of 18 and CLS_9 of 14 (Tables 1, Table S2).

**Table 1:** Lineage and number of cases (N) distributed per year of each IS*6110*-cluster.

Cluster	Lineage	N	2004	2005	2006	2007	2008	2009	2010	2011	2012	2013	2014	2015	2016	2017	2018	2019	2020	SNPs
CLS_2	4.1.2.1	29	3	5	2	**7**	5	1	1	1	2	0	0	0	**2**	0	0	0	0	> 90
CLS_5	4.4.1.1	16	2	2	0	**5**	0	1	0	1	1	0	0	1	**1**	0	0	2	0	≤ 5
CLS_7*	4.8	178	**34**	14	18	22	20	9	12	11	5	4	7	8	3	3	5	**2**	1	< 12
CLS_8	4.3.4.1	19	**6**	2	2	3	1	1	1	1	**1**	1	0	0	0	0	0	0	0	< 5
CLS_9	4.3.3	15	**3**	3	1	2	2	0	0	2	0	0	0	0	**2**	0	0	0	0	14–35
CLS_13	4.8	20	**6**	6	2	1	0	0	1	0	1	1	0	**1**	0	1	0	0	0	≤ 5
CLS_14	4.1.2.1	42	2	**13**	2	4	7	3	3	1	2	**3**	0	0	0	0	0	0	**2**	< 12
CLS_15	4.1.2.1	12	2	**6**	1	1	**1**	0	0	0	1	0	0	0	0	0	0	0	0	> 90
CLS_19	4.9	13	1	**5**	0	1	1	1	1	**2**	0	0	0	0	0	1	0	0	0	≤ 5
CLS_21	4.8	35	0	4	3	**8**	0	0	6	1	1	5	2	2	1	0	**1**	0	1	≤ 5
CLS_24	4.3.2	39	8	**9**	1	3	0	4	1	1	3	1	1	1	0	4	**1**	1	0	< 12
CLS_26	4.7	19	**2**	3	1	1	0	1	0	1	1	1	2	1	0	1	**3**	0	1	> 90
CLS_28	4.8	13	0	**4**	1	1	0	1	0	1	1	0	1	1	**1**	0	1	0	0	≤ 5
CLS_30	4.1.2.1	35	2	2	1	1	**10**	1	3	5	2	2	0	**4**	0	0	0	2	0	≤ 5
CLS_47	4.8	19	**2**	0	2	1	2	1	1	**1**	1	0	2	1	1	1	0	**3**	0	14–35
CLS_49	4.1.2.1	11	0	1	**2**	1	2	0	1	1	**2**	0	0	**1**	0	0	0	0	0	> 90
CLS_50*	4.1.1.3	21	0	**2**	6	0	4	2	1	1	0	1	2	0	0	0	**2**	0	0	< 12
CLS_67	4.1.2.1	19	0	2	0	**3**	3	1	1	0	1	**2**	0	3	**1**	1	1	0	0	≤ 5
CLS_71	4.3.3	19	0	0	1	**2**	3	2	0	0	0	0	3	0	2	1	0	3	**2**	< 12
CLS_91	4.1.2.1	12	0	**1**	0	0	2	0	**3**	3	1	0	1	1	0	0	0	0	0	< 12
CLS_93	4.8	14	0	0	0	0	**3**	2	1	0	0	2	0	0	1	2	0	**3**	0	< 12
CLS_119	4.8	12	0	0	0	0	0	2	0	1	**4**	1	0	0	2	0	**1**	1	0	14–35
CLS_152	4.6.1.1	14	0	0	0	0	0	0	2	1	1	0	**5**	0	0	0	0	2	**3**	≤ 5
CLS_157	4.1.2	10	0	0	0	0	0	0	1	**3**	1	1	1	1	0	0	0	**1**	1	14–35
CLS_195	4.3.2	15	0	0	0	0	0	0	0	0	0	**6**	2	1	5	**1**	0	0	0	≤ 5
CLS_217*	4.3.3	14	0	0	0	0	0	0	0	0	0	0	1	**3**	6	**3**	0	1	0	≤ 5

The bold indicates the year of the selected isolates of each outbreak for this genomic study.

*Cluster independently studied; therefore, a greater number of cases distributed throughout the duration of the outbreak were sequenced. The column “SNPs” shows the SNP distance between the selected isolates of each cluster.

### Regions of Difference (RDs) study

We looked for large deletions (Regions of Difference or RDs)^11^ to find differences between the clustered strains and non-clustered strains previously analysed in our laboratory of the same lineage. According to Coll et al.^12^, RD182 is specific to L4.1.2.1, RD219 is specific to L4.8, RD115 is specific to L4.3.3 and RD724 is specific to L4.6.1.1. All clustered and non-clustered strains were concordant with these specific characteristics. No different RD was found among the clustered and non-clustered strains, with the majority of them being lineage conserved. In addition, the RDs of the isolates belonging to the same IS*6110*-outbreak were the same even in those with a high SNP distance. We only found one different large deletion, not previously described as an RD, between the isolates of CLS_119: one of the isolates had *Rv3054c-Rv3055-dinP-Rv3057* genes deleted, while the other conserved this region as the reference strain. The RDs of the different strains are shown in Table 2.

**Table 2:** RDs of the different IS*6110*-clustered strains studied.

		RD109c	RD115	RD145	RD149	RD152	RD168	RD174 (Rio)	RD178	RD182	RD188	RD193	RD207	RD219	RD252	RD724
L4.1.2	CLS_2			X					X	X						
	CLS_14			X					X	X						
	CLS_15			X					X	X						
	CLS_30			X	X				X	X			X			
	CLS_49			X	X				X	X						
	CLS_67			X					X	X						
	CLS_91			X					X	X						
	CLS_157				X						X					
L4.8	CLS_7					X								X		
	CLS_13										X			X		
	CLS_21													X		
	CLS_28					X	X							X		
	CLS_47					X								X		
	CLS_93													X	X	
	CLS_119					X								X		
L4.3.2	CLS_24				X											
	CLS_195				X	X										
L4.3.3	CLS_9	X	X		X	X			X							
	CLS_71	X	X		X	X			X							
	CLS_217	X	X		X	X			X							
L4.3.4.1	CLS_8				X	X		X								
L4.1.1.3	CLS_50											X			X	
L4.7	CLS26				X	X										
L4.4.1.1	CLS_5					X										
L4.9	CLS_19															
L4.6.1.1	CLS_152															X

X implies the deletion of the genes.

### IS6110 locations

WGS allowed us to locate all the IS*6110* copies in the genomes of the clustered strains. The highest number of copies was found among the strains belonging to L4.3 (average number of IS*6110* copies = 16.3), followed by the L4.8 strains (12.6) and the L4.1.2.1 strains (11.4). The description with the exact locations in all the isolates studied is in Table S3. For the L4.3 strains, three copies were present in all the strains studied—*lpqQ:Rv0836c, Rv1754c* (RD152 area), and *Rv3113*. Moreover, copies located at *cut1*, *ppe38* and *MT3426:MT3427* were frequent. For L4.1.2.1, five copies located at *Rv0403c*, *Rv2336*, *Rv1754c*, *Rv0963c* and *MT3429* were present in all the strains studied. We observed the same locations for other sequenced non-clustered strains of the same lineages (L4.3 and L4.1.2.1). For the L4.8 strains, copies within *MT3429*, *Rv1668:Rv1669c* and *Rv1762c:Rv1763c* (RD152 area) were present in all the strains studied, while copies located at *ppe71* and *Rv0795-Rv0796* were frequent. A summary with the common and frequent IS*6110* copies in the different lineages is shown in Table 3.

**Table 3:** Common and frequent IS*6110* locations in the strains studied, grouped by lineage.

	5’-IS point	3’-IS point	Direct repeat	Gene	Direction	
L4.3	**3,480,373**	**3,480,371**	**cag**	***Rv3113***	**Forward**	100% strains
	**932,202**	**932,204**	**aac**	***lpqQ:Rv0836c***	**Reverse**	
	**1,987,457**	**1,986,625**	**–**	***plcD/Rv1754c***	**Reverse**	
	**1,989,080**	**1,986,625**	**–**	***cut1/Rv1754c***	**Reverse**	
	**1,986,623**	**1,986,625**	**aac**	***Rv1754c***	**Reverse**	
	*1,989,080*	1,979,923*	*cgc*	*cut1*	*Reverse*	81.8% strains
	*1,989,080*	*1,986,625*	*–*	*cut1/Rv1754c*	*Reverse*	
	*2,633,843*	*2,633,841*	*ctc*	*ppe38*	*Reverse*	45.5% strains
	3,665,157*	3,665,159*	*caa*	*MT3426:MT3427*	*Reverse*	91% strains
	3,665,159*	3,668,981*	*–*	*MT3426:MT3427/IS1547*	*Forward*	
L4.8	3,668,619*	3,668,723*	**–**	***MT3429***	**Forward**	100% strains
	**1,895,651**	**1,895,654**	**ccta**	***Rv1668c:Rv1669***	**Reverse**	
	1,986,939*	1,986,937*	**acc**	***Rv1762c:Rv1763***	**Forward**	
	1,979,901*	1,986,937*	**–**	***cut1/Rv1762c:Rv1763***	**Forward**	
	2,604,207*	2,604,210*	*gaaa*	*ppe71*	*Reverse*	85.7% strains
	*889,020*	*890,375*	*gagg*	*Rv0795-Rv0796*	*Forward*	85.7% strains
L4.1.2.1	**1,075,948**	**1,075,950**	**acc**	***Rv0963c***	**Reverse**	100% strains
	**1,986,626**	**1,986,622**	**tgttc**	***Rv1754c***	**Forward**	
	**483,296**	**483,298**	**agg**	***Rv0403c***	**Reverse**	
	**2,610,863**	**2,610,861**	**gcc**	***Rv2336***	**Forward**	
	3,668,575*	3,668,756*	**–**	***MT3429***	**Forward**	

The IS*6110* locations present in all the strains studied belonging to that lineage are in bold, and the frequent locations found are in italic.

*Points referred to the *M. bovis* genome.

We observed some IS*6110* movements among the clustered isolates studied. In five of them, additional copies were detected in the later isolate. In CLS_21, the isolate from 2018 had an extra IS in the DR region that was not present in the isolate from 2007. In CLS_13, the isolate from 2015 had two extra IS copies located in *ppe28:ppe29* and *Rv0756c* that were absent in the 2004 isolate. In CLS_93, the isolate from 2019 had an extra IS copy located in *Rv3177*, absent in the isolate from 2008. In CLS_71, the isolate from 2020 had two extra IS copies located in *Rv1371* and *phoT* that were not present in the isolate from 2007. Finally, in CLS_152, the isolate from 2020 had an extra IS*6110* in *Rv1730c:gabD2* that was absent in the 2014 isolate. On the other hand, in four cases, an extra IS*6110* was detected in the earlier isolate. The CLS_2 isolate of 2007 had an extra IS*6110* located in *Rv3183:Rv3184* that was not in the later isolate of 2016. In CLS_24, the isolate from 2005 had an extra IS*6110* located at *MT3426* that was absent in the 2018 isolate. In CLS 50, the isolate from 2005 had an extra IS copy located in *MT3427*. Lastly, in CLS_91, the isolate from 2005 had an IS*6110* located at the *plcA* gene that was absent in the isolate from 2010. CLS_49 was a special case: the isolate from 2012 had an extra IS inserted within the *Rv1765c* gene (or its homologous *Rv2015c*) that was not present in the other two isolates studied, and the isolate from 2015 had an extra IS located at *ppe49* that was absent in the other two isolates of this cluster. This is in accordance with the large number of SNPs observed among these isolates (143 SNPs). The same could be applied to CLS_2, with an SNP distance of 145. On the other hand, we found identical number of IS*6110* copies and locations for the isolates that apparently were of the same RFLP cluster but, according to the SNP distance, would not be. This occurred for CLS_9, CLS_26, CLS_47, CLS_119 and CLS_157, although the SNP distance for CLS_9 and CLS_119 was very close to 12 (14 and 18 SNPs, respectively). The IS*6110* movements are shown in Table S3.

Some of the deletions observed could be explained by a homologous recombination between two close IS*6110* resulting in the loss of the intermediate genes as one IS*6110* was found interrupting the genes involved. This was observed for CLS_30 isolates, showing the loss of *Rv2817c:Rv2819c* genes; CLS_157 and the deletion of *plcC-plcB-plcA* genes; CLS_13 and the deletion between *plcC* and *ppe40*; CLS_5, CLS_7, CLS_28, CLS_47 and CLS_119, and the deletion between *cut1* and *Rv1765c*; CLS_93 and the deletion between *ppe38* and *ppe71*; CLS_8 and CLS_217, and the deletion of *plcD* gene; CLS_26 and the deletion between *plcD* and *Rv1765c*; CLS_9 and the *Rv2810c:Rv2813* deletion; and CLS_152 and the deletion of *Rv2018* and *Rv2019*.

### SNPs in virulence factors

**Figure 2:**
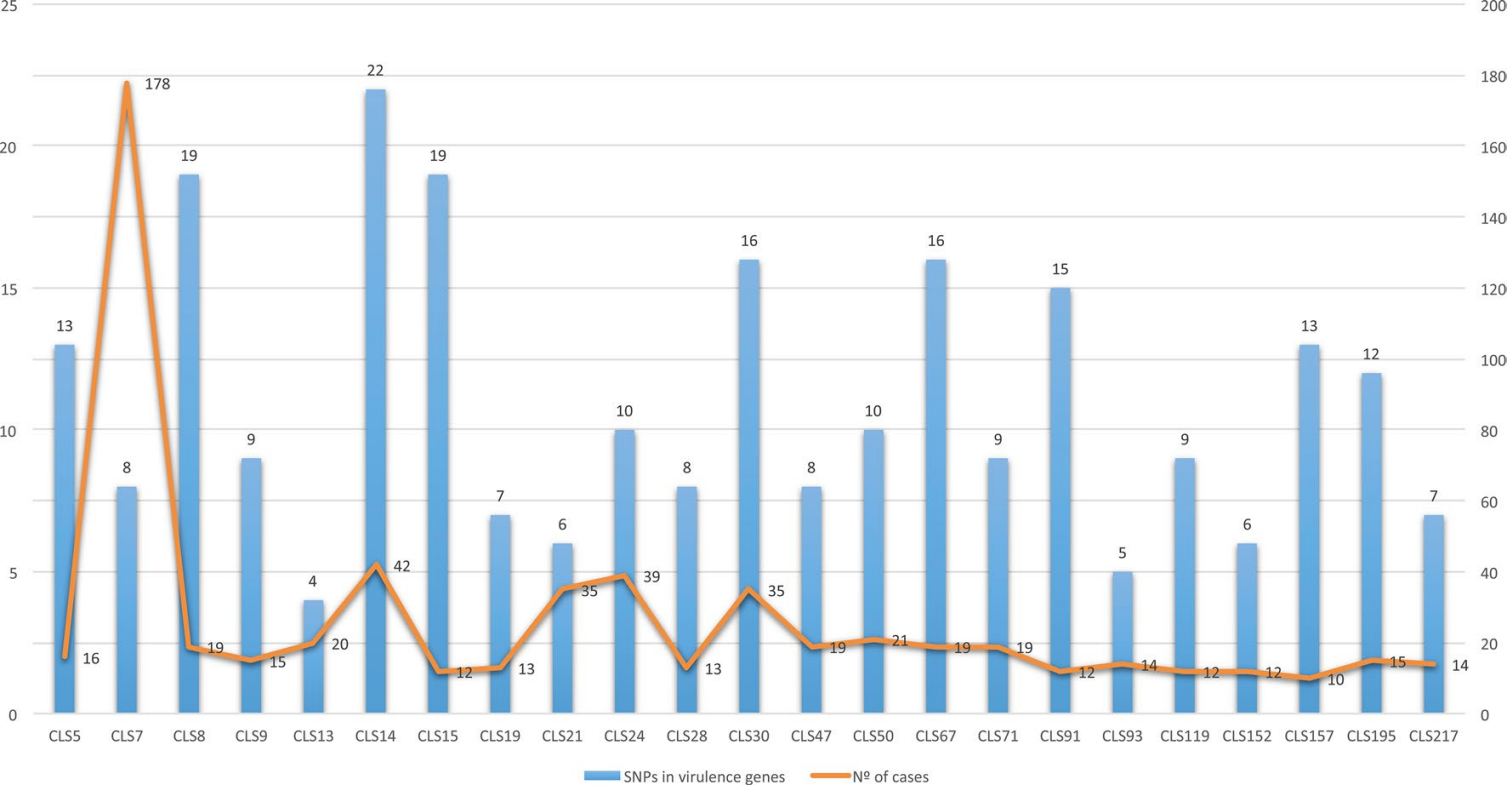
Number of cases of each IS*6110*-cluster vs. SNP number in genes considered as virulence factors. No cause–effect relationship is observed (p-value = 0.8). Clusters with more than 90 SNPs were not considered.

All the L4.1.2.1 strains, including the non-clustered strains reviewed, had 14 common SNPs in genes considered as virulence factors (*mce2D*, *ctpV*, *Rv1290c*, *Rv1505c*, *pks5*, *mgtC*, *secA2*, *Rv1915*, *Rv1915*, *Rv1982c*, *pks12*, *Rv2494*, *cyp125* and *esxB*)^13^. The L4.3 strains had four common SNPs (*mce1D*, *Rv0990c*, *mce3F* and *fadE28*). In addition, there were four more common SNPs for the L4.3.2 strains (*Rv0204c*, *Rv1939*, *Rv3085* and *Rv3871*) and three for the L4.3.3 strains (*mmaA4*, *pks12* and *Rv3088*). The L4.8 strains shared only one common SNP in a gene considered as a virulence factor (*ptpA*). Looking into the isolates studied in the rest of the sub-lineages, the average SNP number was eight, all being unique SNPs. However, there was no relation between the number of SNPs in the genes considered as virulence factors and the number of cases of the IS*6110*-cluster (p-value = 0.8) (Fig. 2).

**Figure 3:**
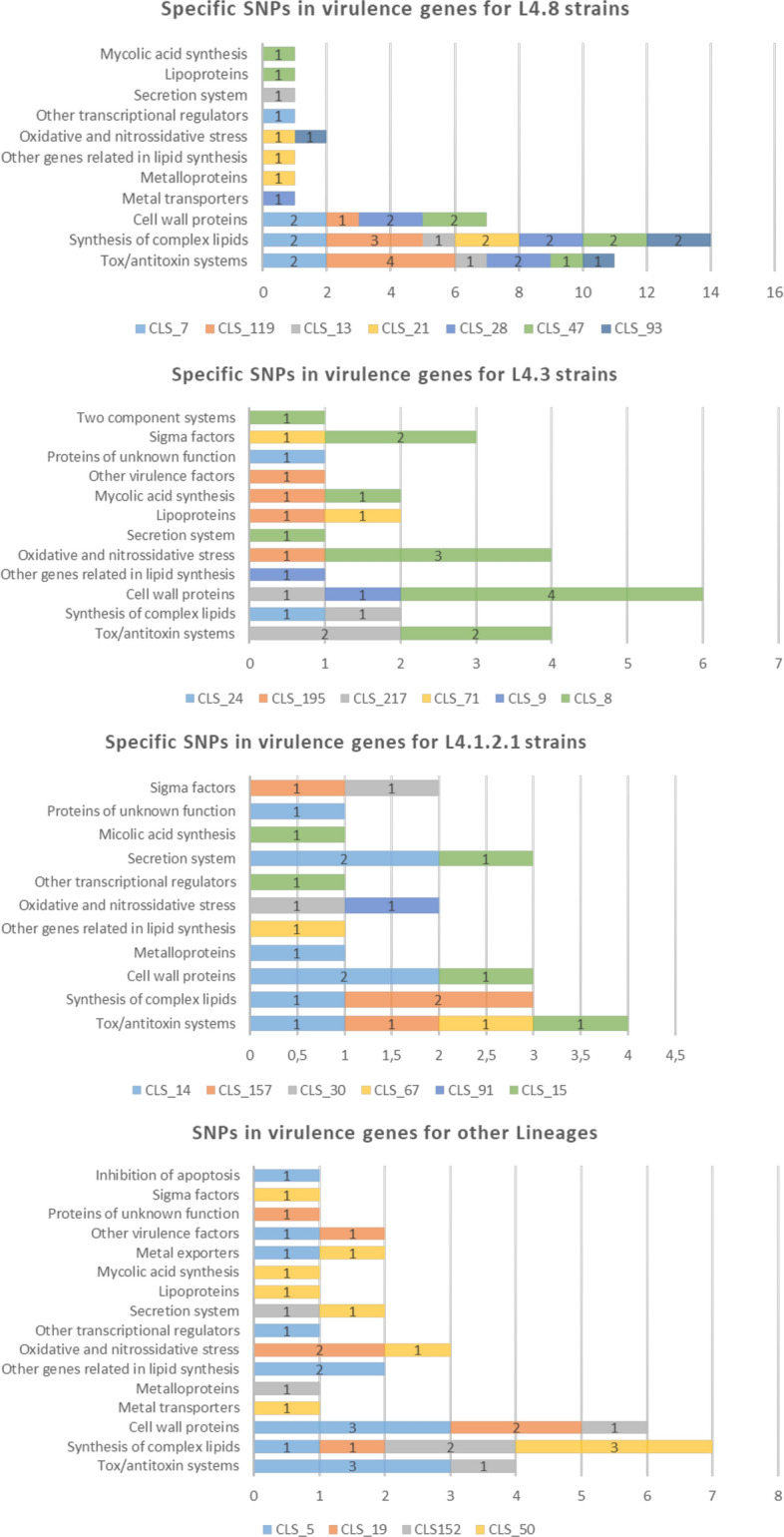
Specific SNPs in virulence genes for each WGS-cluster classified by lineage according to the categories of Forrellad et al. and Ramage et al.^13,14^. The number of categories remains constant among the three main lineages studied (L4.8, L4.3 and L4.1.2.1). The oxidative and nitrossidative stress, other genes related to lipid synthesis, cell wall proteins, synthesis of complex lipids and toxin/antitoxin systems categories are present in the three lineages. For clusters belonging to the L4.4.1.1, L4.9, L4.6.1.1 and L4.1.1.3/X families, there were no common and specific SNPs as only one cluster of each lineage had been sequenced, so comparison was not possible. The categories with more SNPs for these lineages were cell wall proteins, the synthesis of complex lipids and toxin/antitoxin systems. Clusters with more than 90 SNPs were not considered.

As these common sub-lineage SNPs were present in the clustered strains as well as non-clustered strains, we also decided to focus on the specific SNPs of each WGS-cluster strain. The L4.1.2.1 strains turned out to have less specific SNPs (an average of 3.6), followed by the L4.3 (4.7) and L4.8 (5.9) strains. Graphics with the SNPs classified according to the gene categories that Forrellad et al. and Ramage et al.^13,14^ described are shown in Fig. 3. The L4.8 strains had more SNPs in the cell wall proteins (usually *mce* genes), synthesis of the complex lipids and toxin/antitoxin systems categories. For the L4.3 strains, the cell wall proteins category stands out above the rest. For the L4.1.2.1 strains, the secretion systems, cell wall proteins and toxin/antitoxin systems categories have more SNPs. A detailed list of the specific SNPs and descriptions of the genes can be found in Table S4. Regarding the WGS-clusters from other lineages, the synthesis of complex lipids, cell wall proteins and toxin/antitoxin systems categories had more SNPs.

## Discussion

We describe in this work the molecular characteristics of *M. tuberculosis* strains that produced the largest outbreaks in our community. Three of these outbreak strains were previously characterised^7–9^ (CLS_ 217, CLS_50 and CLS_7). The majority of publications about TB outbreaks based on WGS focus on the power of this methodology to determine whether the isolates belong to the same cluster and/or attempt to elucidate the transmission chain^10,15^. However, few articles focus on the molecular characteristics of relevant strains using WGS^16^.

A total of 665 TB cases had been caused by some of these studied strains since 2004, which represents 26% of the total TB cases in our community. As expected, all these strains belonged to L4, which is the lineage responsible for the majority of TB cases in our population^4^. The most widespread lineages were L4.8, L4.1.2.1/Haarlem and L4.3/LAM, in concordance with the dominant lineages in Spain described by Stucki et al.^4^.

Regarding the SNP distance, the WGS results were in agreement with the RFLP-IS*6110* patterns for 18 outbreaks, including the three previously studied ^7–9^: the genetic distance was between 1 and 10 SNPs, so they were considered epidemiologically linked isolates^10,17,18^. However, the classical gold-standard RFLP technic failed to cluster the isolates of CLS_2, CLS_49 and CLS_26. For CLS_2, there was an extra IS*6110* in one of the isolates that was not captured in the RFLP patterns. For CLS_49, two isolates had an identical RFLP pattern, while the third had an extra band, although initially, it was considered the same strain that had evolved. WGS confirmed this extra IS*6110* for this isolate and a different one for the other isolates that RFPL did not show. As for CLS_26, the RFLP pattern was the same but also the IS*6110* locations found by WGS, a total of six, which is the threshold indicated for discrimination by RFLP pattern. Thanks to the greater resolution power of WGS^19^, we deciphered that these IS*6110*-clustered isolates were not epidemiologically related as the SNP distance was more than 90 despite sharing similar IS locations.

On the other hand, WGS did not clarify the relatedness in CLS_9, CLS_47, CLS_119 and CLS_157 using only the SNP distance information. These IS*6110*-clusters have distances of more than 12 SNPs but are very close. Furthermore, the IS*6110* locations found by WGS were exactly the same, along with their RFPL patterns (16, 13, 12 and 11 IS*6110*, respectively). The number of bands is large/high enough to be considered a fluke, reflecting that both isolates corresponded to the same strain despite the number of SNPs being ≥ 12. There is a fact about CLS_119 that would support the idea of separating one isolate from its pair: the deletion of the *Rv3054*:*Rv3058* region in one of the isolates but not in the other. We considered this deletion, which could not be explained by an IS*6110* recombination, an independent event that took place in this particular isolate. It is important to consider that the traceability between the selected isolates is not defined, making the intermediate cases that have occurred between them unknown. Although this is not frequent, strains can evolve during the transmission process. If the isolates of CLS_119 are not epidemiologically linked, a genetic separation between them must have taken place recently. It would be interesting to analyse the complete isolates of this cluster to understand their evolution, as well as the rest of the clusters to track for recent transmission. However, the 12 SNP threshold should be polished to clarify these intermediate SNP distances as for now, the significance of their relatedness is unclear.

One important fact is that the mutation rate is not the same for all MTBC strains. In the independent studies we made for CLS_7 and CLS_50^7,9^, we found that all the isolates studied had ≤ 12 SNPs with at least one isolate, confirming its belonging to the outbreak, although the strains had been circulating for more than 25 years. Some isolates presented several unique SNPs, which could be due to sequencing errors or favoured by the epidemiological characteristics of the patient, which could increase the SNP distance. For the outbreaks studied in this work, we only analysed two or three isolates; therefore, the SNP distance with some other isolates of the IS*6110*-cluster could be ≤ 12, indicating its membership to the outbreak. This could be the case of CLS_9, with an SNP distance of 14.

The RD study showed that the majority of RDs are lineage conserved, so they are present in clustered as well as non-clustered strains. Nevertheless, RD152 and RD188 are distributed among different sub-lineages, which may be due to both regions containing hot spots for IS*6110* insertions, causing later recombination events^20–22^. RD178 (a fragment of the *helZ* gene), RD252 and RD149 are also distributed among different lineages, especially RD149. This deletion has been associated with a reduction in growth and an increase in the induction of TNFα in host cells by CAS1 strains^23^. However, as this RD is also present in the non-clustered strains, this may not be responsible for the success in transmission of the outbreak strains.

WGS allowed an easier study of the IS*6110* location than the molecular techniques based on PCR, previously used. We found some lineage-conserved locations, in accordance with those described before^24^. It called our attention to the presence of an IS*6110* copy in the *ppe38/ppe71* locus in many of the strains studied (67.4%). The deletion of this locus has been related to virulence in L2/Beijing strains^25^, so the disruption of these genes by an IS*6110* could be an advantage for the mycobacteria. This could be the reason why so much polymorphism is observed in this region. The study of the IS also allowed us to discover movements of this mobile element between the different isolates for 10 of the clusters studied. Some of them were captured by the RFLP pattern, but others were not. These observations lead us to believe that IS*6110* transposition could occur during infection, although majority of the time, it would fail, provoking some handicap to the bacteria.

We decided to study SNPs in genes considered virulence factors as we previously observed that a single SNP in one of these genes could be responsible for higher transmission^7,9^. We focused on the specific SNPs of each cluster strain as the ones present in both clustered and non-clustered strains would not be related to higher virulence or transmission. We observed no relationship between the number of SNPs in these genes and the number of cases the strain produced, so we concluded that a particular SNP is more important than the number. We describe these SNPs in Table S4, but more research is required to determine whether some of them are responsible for the increase of transmission of these outbreak strains. SNPs in the categories oxidative and nitrossidative stress, other genes related in lipid synthesis, cell wall proteins, synthesis of complex lipids and toxin/antitoxin systems are present in almost all the strains studied, so those genes may be important for the success of the outbreak strains.

In conclusion, we describe in this work the molecular characteristics—lineage, presence or absence of RD, IS*6110* locations and SNPs in virulence factors—of the most successful strains in our population. We give value to the classical techniques maintained along the time to track the pathway of these strains. We are deep into evolution and have identified potential outbreak features, shared by some of these strains, to develop surveillance actions.

## Materials and methods

### Clinical sample selection

All IS*6110*-RFLP and Spoligo patterns of the *M. tuberculosis* isolates from 2004 to 2020 were loaded in the Bionumerics database (v7.6, Applied Maths, Kortrijk, Belgium). Among the 2553 genotyped isolates, we selected two or three isolates belonging to each of the IS*6110*-clusters involving 10 or more cases of TB: CLS 2 (one isolate from 2007 and one from 2016), CLS 5 (2007/2016), CLS 7 (2004/2019), CLS 8 (2004/2012), CLS 9 (2004/2016), CLS 13 (2004/2015), CLS 14 (2005/2013/2020), CLS 15 (2005/2008), CLS 19 (2005/2011), CLS 21 (2007/2018), CLS 24 (2005/2018), CLS 26 (2004/2018), CLS 28 (2005/2016), CLS 30 (2008/2015), CLS 47 (2004/2011/2019), CLS 49 (2006/2012/2015), CLS 50 (2005/2018), CLS 67 (2007/2013/2016), CLS 71 (2007/2020), CLS 91 (2005/2010), CLS 93 (2008/2019), CLS 119 (2012/2018), CLS 152 (2014/2020), CLS 157 (2011/2019), CLS 195 (2013/2017) and CLS 217 (2015/2017). Three of these clusters were independently studied by WGS: CLS 7^7^ (57 isolates sequenced), CLS 50^9^ (32 isolates sequenced), and CLS 217^8^ (13 isolates sequenced).

Thirteen genomes from different sub-lineages, six from L4.1.2 and seven from L4.3, not involved in any of the large outbreaks (non-clustered strains) and sequenced in previous studies, were used to make the comparisons with the clustered strains belonging to these sub-lineages.

### DNA extraction and classical genotyping

DNA was extracted from bacterial cultures using the cetrimonium bromide method described by van Soolingen^26^. The IS*6110*-RFLP and Spoligo genotyping were made for all the isolates, as previously described^27,28^. An IS*6110*-cluster was defined as having the same or similar RFLP pattern (one extra IS*6110* band was accepted if some epidemiological link was found). Mycobacterial interspersed repetitive unit-variable number of tandem repeats (MIRU-VNTR) was performed for one isolate of each cluster^29^. The DNA samples were stored at − 20 °C until sequencing.

### Whole-genome sequencing (WGS)

The majority of DNA (50 isolates) were sequenced using Illumina technology. However, some DNA used in previous studies (six isolates) were sequenced using the IonTorrent sequencing platform. Both technologies were applied according to the manufacturer’s instructions. After sequencing, the fastQ files obtained were mapped against the reference strain H37Rv (NC_000962.3) to obtain the Binary Aligned Map (bam) and the Variant Call Format (vcf) files. The SNP classification established by Coll et al.^12^ was used for identifying the MTBC lineage of the outbreak strains. This classification is based on the specific SNPs of the different lineages.

### Bioinformatics

The Bionumerics software was used for the SNP study and for the construction of the dendrograms using the UPGMA method. For greater accuracy, strict SNP filtering that removed positions with at least one ambiguous or unreliable base, gaps (maximun frequency 1%), non-discriminatory positions and *ppe* and *pgrs* genes, was applied. It was also considered that the retained SNP positions had a minimum 5 × coverage and that the minimum distance between SNPs was at least 12 base pairs (bp). The Integrative Genomics Viewer (IGV, from the Broad Institute^30^) software was used for the RD study and the SNP study. GeneWise (https://www.ebi.ac.uk/Tools/psa/genewise/) and PROVEAN (http://provean.jcvi.org/seq_submit.php) platforms were used for the SNP study, predicting whether an SNP is synonymous or non-synonymous and the effect of a non-synonymous mutation is neutral or deleterious, respectively. All SNPs are referred to H37Rv genome (NC_000962.3), unless otherwise indicated.

From the fastQ files, the reads containing the first 30 bases of the IS*6110* and the ones with the last 30 were extracted. We used Tuberculist (http://genolist.pasteur.fr/TubercuList/) and Bovilist (http://genolist.pasteur.fr/BoviList/) to apply BLAST and find the IS*6110* insertion points. Mycobrowser (https://mycobrowser.epfl.ch/) and UniProtKB (https://www.uniprot.org/uniprot/) websites were used to find information on the genes and proteins with noteworthy SNPs.

## Supplementary Information

Supplementary Table S1.

Supplementary Table S2.

Supplementary Table S3.

Supplementary Table S4.

## Data Availability

The fastq files of the selected isolates of each outbreak are uploaded in GenBank with the accession numbers SAMN26722357-SAMN26722406 (BioProject accession number PRJNA816739). https://www.ncbi.nlm.nih.gov/bioproject/?term=PRJNA816739.
